# Inhibition of *Streptococcus mutans* adhesion and biofilm formation with small-molecule inhibitors of sortase A from *Juniperus chinensis*

**DOI:** 10.1080/20002297.2022.2088937

**Published:** 2022-06-14

**Authors:** Eunji Cho, Ji-Yeon Hwang, Jae Sung Park, Daehyun Oh, Dong-Chan Oh, Hyeung-Geun Park, Jongheon Shin, Ki-Bong Oh

**Affiliations:** a Department of Agricultural Biotechnology, College of Agriculture and Life Sciences, Seoul National University, Seoul, Republic of Korea; b Natural Products Research Institute, College of Pharmacy, Seoul National University, Seoul, Republic of Korea; c Research Institute of Pharmaceutical Sciences and College of Pharmacy, Seoul National University, Seoul, Republic of Korea

**Keywords:** Adhesion, biofilm formation, *Juniperus chinensis*, sortase A inhibitor, *Streptococcus mutans*

## Abstract

**Background:**

*Streptococcus mutans*, an important Gram-positive pathogen in dental caries, uses sortase A (SrtA) to anchor surface proteins to the bacterial cell wall, thereby promoting biofilm formation and attachment to the tooth surface.

**Design:**

Based on activity-guided separation, inhibitors of *S. mutans* SrtA were isolated from *Juniperus chinensis* and identified through combined spectroscopic analysis. Further effects of isolated SrtA inhibitor on *S. mutans* were evaluated on bacterial aggregation, adherence and biofilm formation.

**Results:**

Six compounds (**1–6**) were isolated from the dried heartwood of *J. chinensis*. A novel compound designated 3’,3”-dihydroxy-(−)-matairesinol (**1**) was identified, which exhibited potent inhibitory activity toward *S. mutans* SrtA (IC_50_ = 16.1 μM) without affecting microbial viability (minimum inhibitory concentration > 300 μM). The results of subsequent bioassays using compound **1** indicated that this compound inhibits *S. mutans* aggregation, adhesion and biofilm formation on solid surfaces by inhibiting SrtA activity. The onset and magnitude of inhibition of adherence and biofilm formation in *S. mutans* treated with compound **1** at 4× the SrtA IC_50_ are comparable to the behaviors of the untreated *srtA*-deletion mutant.

**Conclusion:**

Our findings suggest that small-molecule inhibitors of *S. mutans* SrtA may be useful for the prevention of dental plaque and treatment of dental microbial diseases.

## Introduction

Gram-positive pathogenic bacteria have many surface proteins related to bacterial adherence and host invasion, which play key roles in virulence. Sortase A (SrtA) is a transpeptidase that controls anchoring of surface proteins in the peptidoglycan cell walls of Gram-positive bacteria [1]. This protein recognizes the LPXTG motif in substrates, severs the amide bond between T and G residues, and forms a covalent bond between the substrate and cell wall [2]. Because numerous Gram-positive bacteria possess genes encoding SrtA and surface proteins with sorting signals recognized by SrtA, the SrtA-mediated anchoring system is considered a universal mechanism [3]. Knockout mutants of *srtA* fail to display surface proteins with the LPXTG motif which leads to diminished infectiousness without impacting bacterial viability [4–6]. For example, *Staphylococcus aureus srtA* mutants were not able to display Spa (protein A), FnbA (fibronectin-binding protein), ClfA (clumping factor) proteins, and showed impaired infections in mice [4]. Due to these properties, SrtA is closely associated with the virulence of Gram-positive pathogens and is considered a desirable anti-virulent drug target [7].

*Streptococcus mutans* is a facultative aerobic Gram-positive bacterium and an important cariogenic pathogen. This bacterium inhabits the human oral cavity, causing dental plaque and dental caries [8]. The major virulence factors of *S. mutans* are the capacity to form biofilms attached to the tooth surface, the capacity to produce organic acids (acidogenicity), and viability in low-pH conditions (aciduricity) [9]. Biofilm formation by *S. mutans* proceeds via sucrose-dependent and sucrose-independent pathways, following the steps of adherence, aggregation, microcolony formation, and maturation [10]. Research using *srtA* knockout mutant strains of *S. mutans* revealed the importance of SrtA in these processes: *srtA* mutant could not anchor surface proteins and showed less adherence and aggregation than wild-type strain [11]. Because the attachment and aggregation are early steps of biofilm formation, the mutants exhibited a lower tendency to colonize surfaces of the oral mucosa or teeth, as well as reduced biofilm accumulation [12]. Considering the close relationship between sorting the surface proteins, biofilm formation, and virulence in *S. mutans*, SrtA is a promising anti-virulent target like other Gram-positive bacteria [7].

Based on an initial screening of bioactive metabolites that may function as SrtA inhibitors, extracts of 120 commercially available Korean traditional medicines were tested against *S. aureus-* and *S. mutans*-derived SrtA. Some of these extracts showed enzyme inhibitory activity exceeding 40% at 100 μg/mL concentration. The SrtA inhibitory metabolites of these traditional medicines were identified as curcuminoids from *Curcuma longa* [13], flavonoids from *Sophora flavescens* [14], lignans and phenyl propanoids from *Pulsatilla koreana* [15], maltol derivatives and flavonol glycosides from *Sophora japonica* [16,17], flavonoids from *Psoralea corylifolia* [18] and *Spatholobus suberectus* [19,20], and coumarins from *Poncirus trifoliata* [21].

**Figure 1.**
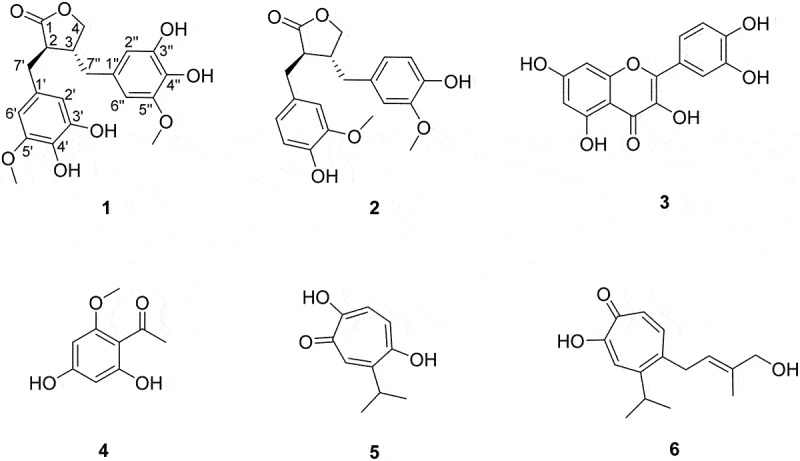
Structures of compounds **1–6** from *Juniperus chinensis*: 3’,3”-dihydroxy-(−)-matairesinol (**1**), (−)-matairesinol (**2**), quercetin (**3**), 4,6-dihydroxy-2-methoxyacetophenone (**4**), 5-hydroxyhinokitiol (**5**), and juniperone A (**6**).

In this study, chemical structures were investigated in *Juniperus chinensis* crude extract, which effectively inhibited *S. mutans*-derived SrtA (71.2% inhibition at a concentration of 100 μg/mL). Bioassay-guided fractionation of the extract using various chromatographic methods following combined spectroscopic analysis yielded six compounds (**1–6**) of various skeletal classes including, flavonoid, lignan, and tropolone-bearing sesquiterpene (Figure 1). Among these compounds, a novel lignan (**1**) was structurally characterized as a dihydroxy derivative of matairesinol through combined spectroscopic analysis. This study describes the structures of a novel lignan (**1**) and several previously reported compounds (**2**–**6**) isolated from *J. chinensis*. Compound **1** strongly inhibited *S. mutans*-derived SrtA. The magnitudes of inhibition of aggregation, adhesion, and biofilm formation in *S. mutans* treated with compound **1** is comparable to the behaviors of the untreated *srtA*-deletion mutant. These results suggest that an inhibitor of SrtA may be a useful tool for inhibiting the cariogenic properties of *S. mutans*.

## Materials and methods

### General experimental procedures

Optical rotation was measured with a JASCO P-2000 polarimeter (Easton, MD) with a 1-cm cell. Ultraviolet (UV) spectra were acquired using a Hitachi U-3010 spectrophotometer (Tokyo, Japan). Infrared (IR) spectra were obtained with a JASCO 4200 FT-IR spectrometer (Easton, MD) using a ZnSe cell. High-resolution electrospray ionization mass spectrometry (HR-ESI-MS) data were acquired at the National Instrumentation Center for Environmental Management (Seoul, Korea) using an AB Sciex 5600 QTOF HR-MS instrument (Sciex, MA). Proton and carbon nuclear magnetic resonance spectroscopy (NMR) and two-dimensional NMR spectra were recorded with a Varian Gemini 2000 300 MHz spectrometer (Palo Alto, CA) or Bruker Avance 500 and 600 MHz spectrometers (Berlin, Germany) using MeOH-*d*_4_ with a solvent peak at δ_H_ 3.31/δ_C_ 49.0 or dimethyl sulfoxide (DMSO)-*d*_6_ with a solvent peak at δ_H_ 2.50/δ_C_ 39.50 as internal standards. High-performance liquid chromatography (HPLC) separation was conducted on a SpectraSYSTEM p2000 instrument equipped with a refractive index detector (SpectraSYSTEM RI-150) and a UV-Vis detector (UV-Vis-151, Gilson, Middleton, WI). All solvents used were of spectroscopic grade or were distilled prior to use.

### Plant material

The dried heartwood of *J. chinensis* was purchased from Kyungdong Oriental Herbal Market in Seoul, Korea, on 13 November 2017. A voucher specimen (specimen number 2017-YH-1) was deposited at the Natural Products Research Institute, College of Pharmacy, Seoul National University, Seoul, Republic of Korea. Taxonomic identification of the specimen was conducted by prof. Y. Suh at Seoul National University.

### Extraction and isolation of compounds

The macerated heartwood (5.0 kg) of *J. chinensis* was repeatedly extracted into methanol (5 L × 3) and dichloromethane (5 L × 3) at room temperature. The organic extracts were combined and concentrated in a rotary evaporator. The residue (247.1 g) was fractionated between water and *n*-butanol, and then the latter layer (223.1 g) was partitioned between water–methanol (15:85) and *n*-hexane. The water–methanol portion (117.8 g) was separated into eight fractions through C_18_ reversed-phase vacuum flash chromatography and eluted with sequential mixtures of methanol and water (six fractions across a water–methanol gradient from 50:50 to 0:100) followed by acetone and finally ethyl acetate.

According to the results of ^1^H NMR analysis, the fractions eluted with water–methanol (50:50) and (40:60) were separated further. The water–methanol (50:50) fraction (32.7 g) was subjected to semi-preparative reversed-phase HPLC (YMC-ODS column, 250 × 10 mm; 2.0 mL/min; water–methanol, 68:32). The peak at *t*_R_ = 43.3 min was purified through analytical HPLC (0.7 mL/min; water–acetonitrile gradient from 80:20 to 65:35 over 60 min) to yield compound **1** (*t*_R_ = 47.5 min). The fraction (23.4 g) eluted with water–methanol (40:60) was separated through HPLC (1.8 mL/min; water–methanol, 50:50) to obtain compounds **2** (*t*_R_ = 33.2 min) and **3** (*t*_R_ = 46.5 min). Purification of an additional peak (*t*_R_ = 44.7 min) through analytical HPLC (0.7 mL/min; water–acetonitrile, 75:25) produced compounds **4** (*t*_R_ = 6.8 min) and **5** (*t*_R_ = 60.1 min). The peak at *t*_R_ = 70.8 min was purified through analytical HPLC (0.7 mL/min; water–acetonitrile, 70:30) to obtain compound **6** (*t*_R_ = 46.3 min). The overall isolated amounts of compounds **1–6** were 13.2, 3.6, 3.3, 4.2, 4.8, and 1.1 mg, respectively.

*Chemical characteristics of 3’,3”-Dihydroxy-(*−*)-matairesinol (**1**)*: yellow, amorphous solid; specific rotation at 25°C [α] −28.1 deg cm^2^/g (0.4 g/100 ml in MeOH); wavelengths of maximum absorbance and log value of molar absorptivity on UV spectra in MeOH λ_max_ (log *ε*) 231 (3.36), 272 (2.86) nm; band maxima of frequency on IR spectra on the ZnSe cell ν_max_ 3276, 1760, 1607, 1515, 1456, 1340, 1204, 1151, 1093, 1022 cm^−1; 1^H and ^13^C NMR data are presented on Table 1 (^1^H and ^13^C NMR spectrum: Figures S1 and S2, respectively); HR-ESI-MS mass number/charge number of ion (*m/z*): 391.1388 [M + H]^+^ (calculated for C_20_H_23_O_8_, 391.1387) (Figure S7).

**Table 1.** The ^13^C and ^1^H NMR data of compound **1** (δ_H_ and δ_C_ in ppm)

	1^a^	
No.	δ_C_, type	δ_H_ (*J* in Hz)
1	178.5, C	
2	45.6, CH	2.63, m
3	40.8, CH	2.39, m
4	70.7, CH_2_	4.03, t (8.0)
		3.83, t (8.0)
1’	128.1, C	
2’	104.7, CH	6.26, d (2.0)
3’	148.2, C	
4’	132.4, C	
5’	145.6, C	
6’	109.9, CH	6.31, d (2.0)
7’	33.9, CH_2_	2.74, dd (13.5, 5.5)
		2.69, dd (13.5, 6.5)
OCH_3_	55.7, CH_3_	3.71, s
1”	128.9, C	
2”	103.8, CH	6.15, d (2.0)
3”	148.4, C	
4”	132.6, C	
5”	145.7, C	
6”	109.2, CH	6.17, d (2.0)
7”	37.2, CH_2_	2.44, dd (12.5, 9.0)
		2.32, dd (13.0, 5.0)
OCH_3_	55.7, CH_3_	3.70, s

^a^Data were obtained in DMSO-*d*_6._

### SrtA inhibition assay

Recombinant SrtA protein was prepared following previously described procedures [16,22]. *S. mutans* OMZ65 isolated from the human oral cavity was provided by Seoul National University School of Dentistry. The *srt*A genes derived from *S. mutans* OMZ65 were expressed in *Escherichia coli* and SrtA was purified through metal chelate affinity chromatography on Ni-nitriloacetic acid (NTA) resin. Each reaction was performed in a total volume of 100 μL, containing 50 mM Tris-HCl, 150 mM NaCl, and 5 mM CaCl_2_ at pH 7.5, along with 17 μg of purified SrtA and 250 ng of synthetic substrate (Dabcyl-LPETG-Edans). The sample compounds were added to each reaction mixture with the solvent DMSO (final concentration, 1%). After 1 h of incubation at 37°C, the reactions were quantified based on fluorometric intensity (350 nm excitation and 495 nm emission wavelengths) using a microplate reader (FLx800, BioTek Instruments, Winooski, VT). Triphasiol was used as a positive control for the SrtA inhibitor.

### Antibacterial activity assay

Antibacterial activities of isolated compounds were determined according to a previously described method [23] based on Clinical and Laboratory Standards Institute guidelines [24]. Briefly, 5 mL of *S. mutans* OMZ65 was inoculated into brain heart infusion (BHI) broth, incubated aerobically for 16 h at 37°C, and the bacterial density was adjusted based on turbidity to match the 0.5 MacFarland standard at 625 nm. In each well of a 96-well plate, 20 μL of twofold diluted test compounds in 10% DMSO was added to 180 μL of cell culture in BHI broth and incubated for 16–20 h at 37°C. The final concentration of cells was approximately 5 × 10^5^ colony-forming units (CFU)/mL. The minimal inhibitory concentration (MIC) values were identified as the lowest concentration that inhibited cell growth. Ampicillin was used as an antibacterial positive control.

### Saliva-induced aggregation assay

The effect of SrtA inhibitor on bacterial cell aggregation induced by human saliva was conducted according to a previously documented procedure [11]. *S. mutans* NG8 (wild-type), *srtA*-deletion mutant (*ΔsrtA*), and *srtA*-complemented mutant (*ΔsrtA+srtA*) were used in this study. These strains were kindly provided by Prof. S. F. Lee (Dalhousie University, Nova Scotia, Canada) and inoculated into Todd Hewitt broth and incubated for 16 h at 37°C, washed twice with KPBS (137 mM NaCl, 2.7 mM KCl, 6.5 mM Na_2_HPO_4_, 1.5 mM KH_2_PO_4_, pH 7.2), and resuspended in KPBS to an approximate optical density of 1.0 at 700 nm. Assay mixtures consisted of 400 μL of cells, 100 μL of fresh clarified saliva, and diluted test compounds. Saliva was provided from one volunteer (a 30-year-old unmarried woman) who had no dental health issues. The collected non-stimulated saliva was clarified by centrifugation (7,000 × g, 10 min, 4°C) and sterilized by membrane filtration (0.2 μm pore size). The mixtures were gently mixed through inversion and incubated aerobically at 37°C for 2 h. Turbidity at 700 nm was recorded every 20 min.

### Adherence assay on saliva-coated hydroxyapatite beads

The effect of SrtA inhibition on bacterial adherence was evaluated using saliva-coated hydroxyapatite beads (s-HAs) [11,25]. A total of 30 μg of s-HAs (diameter, 80 µm; Bio-Rad, Hercules, CA) were coated with fresh saliva prepared as mentioned above for 1 h at room temperature and rinsed twice with 0.01 M potassium phosphate buffer (KPB; pH 7.0). *S. mutans* NG8 (wild type), *srtA*-deletion mutant (*ΔsrtA*), and *srtA*-complemented mutant (*ΔsrtA + srtA*) [11] were inoculated into 5 mL of BHI broth, incubated aerobically for 16 h at 37°C, and diluted to about 10^8^ CFU/mL. The cell suspension and s-HAs were mixed with or without compound **1** (final concentration of cells 10^7^ CFU/mL with 1% DMSO) and incubated for 90 min at 37°C with weak shaking. After three rounds of washing with KPB, attachment between s-HAs and *S. mutans* cells was disrupted through sonication (50 W, 30s) in KPB. Dispersed cells were spread on Mitis-Salivarius agar (Difco, Detroit, MI) plates containing 3.2 mg/mL bacitracin. The number of colonies formed was determined after incubation for 48 h at 37°C.

### Biofilm formation assay

The effect of SrtA inhibition on bacterial biofilm formation was investigated using the polystyrene plate and resin teeth model according to a previously described method [12]. Wild-type *S. mutans* and *srtA* mutants were inoculated aerobically into 5 mL of BHI broth at 37°C. Cultures in BHI broth containing 0.1% sucrose (final concentration 5 × 10^5^ CFU/mL) were incubated with or without test compounds on 96-well polystyrene plates or with resin teeth (Endura, Shofu Inc., Kyoto, Japan) at 37°C for 24 h under aerobic condition. Biofilms formed on the plate and resin teeth were washed twice with distilled water and then stained with 0.1% safranin for 30 s. After three further washes, the safranin-bound biofilms on plates were dissolved with 30% acetic acid (v/v aqueous solution) and the intensity of absorbance at 530 nm wavelength, indicating safranin, was measured. Biofilms on resin teeth was also washed three times and photographed.

### Statistical analysis

Statistical analysis was conducted using GraphPad Prism 9.3.1 software (Graphpad, San Diego, CA). Statistical differences between groups were analyzed with Student’s *t*-test or two-way analysis of variance followed Dunnett’s test for post-hoc analysis. A *p*-value < 0.05 was regarded as statistically significant.

## Results

### Structural characterization of compounds 1–6

The molecular formula of compound **1** was determined to be C_20_H_22_O_8_ through HR-ESI-MS analysis ([M + H]^+^ *m/z* 391.1388, calculated for C_20_H_23_O_8_, 391.1387) (Figure S7). Using heteronuclear single quantum coherence NMR data, the ^13^C and ^1^H NMR features of this compound were identified as a carbonyl carbon (δ_C_ 178.5), 12 aromatic methines and non-protonated carbons (δ_C_/δ_H_ 148.4–103.8/6.31–6.15), two methoxy groups (δ_C_/δ_H_ 55.7/3.71 and 55.7/3.70), three methylenes (δ_C_/δ_H_ 70.7/4.03 and 3.83, 37.2/2.44 and 2.32, and 33.9/2.74 and 2.69), and two methines (δ_C_/δ_H_ 45.6/2.63 and 40.8/2.39) (Table 1, Figures S1 and S2). These characteristic features of 18 carbons (excluding the two methoxy groups) including a carbonyl and 12 aromatic carbons, in conjunction with an unsaturation degree of 10 determined from the mass data, strongly indicated that compound **1** is a lactone-bearing lignan compound.

**Figure 2.**
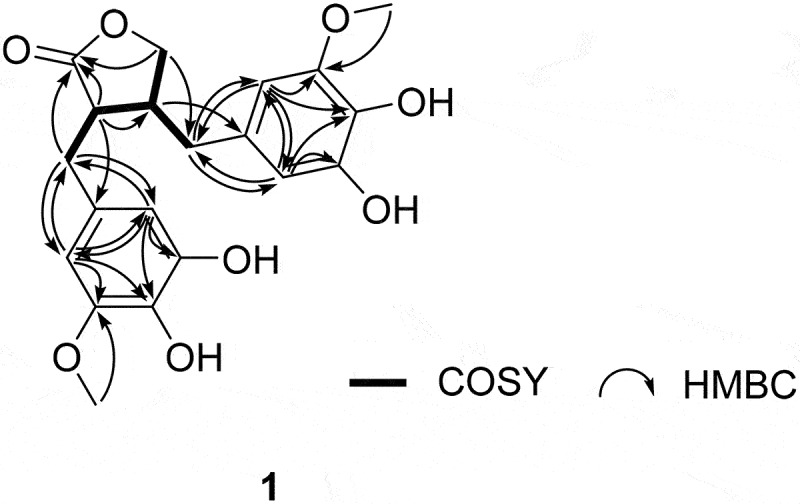
Key correlations found in the ^1^H correlated spectroscopy (bold) and heteronuclear multiple bond correlation (arrows) experiments for compound **1.**

Based on this information, the planar structure of **1** was determined from a combination of ^1^H-^1^H coupling constants, ^1^H correlated spectroscopy, and heteronuclear multiple bond correlation NMR analyses (Figure 2, Figures S3-S6). The three key structural motifs were readily identified as 2,3-dimethylene bearing butyrolactone (C-1-C-4, C-7’, and C-7”) and two 3,4-dihydroxy-5-methoxyphenyl groups (C-1’-C-6’ and C-1”-C-6”) based on the conspicuous ^1^H-^1^H and ^1^H-^13^C correlations. Subsequently, the connections of aromatic moieties at the lactone methylenes were assessed using a series of long-range ^1^H-^13^C correlations: H-2/C-1’, H-3/C-1”, H-2’/C-7’, H-6’/C-7’ H_2_-7’/C-2’ and C-6’, H-2”/C-7”, H-6”/C-7”, and H_2_-7”/C-2” and C-6”. Literature review revealed that the deduced planar structure of **1** was the 3’,3”-dihydroxy derivative of matairesinol, a dibenzylic butanolide lignan found in *Cryptomeria japonica* [26] and *Forsythia koreana* [27].

Compound **1** possessed two stereogenic centers at C-2 and C-3 of the lactone moiety. As extensively described in the literature [28,29], the non-equivalence of H_2_-4 (δ_H_ 4.03 and 3.83) assured the *trans* configuration between C-2 and C-3. Then, the absolute configurations were assigned as 2 *R* and 3 *R* based on comparison of its specific rotation ([α] −28.1) with those of (−)- and (+)-matairesinol ([α] and −47.2 and +15.2 for the (−) and (+) enantiomers, respectively) [30,31]. Thus, the structure of compound **1**, designated 3’,3”-dihydroxy-(−)-matairesinol, was determined to be a novel 2,3-dibenzyl-4-butanolide lignan.

In addition to compound **1**, five congeners of diverse structural classes were isolated and identified through combined spectroscopic analysis and literature comparison: (−)-matairesinol (**2**), quercetin (**3**) [32], 4,6-dihydroxy-2-methoxyacetophenone (**4**) [33], 5-hydroxyhinokitiol (**5**) [34], and juniperone A (**6**) [35]. Spectroscopic data for these compounds were in accordance with information in the literature (Tables S1 and S2).

### SrtA inhibitory activities of compounds 1–6

Recombinant SrtA protein derived from *S. mutans* OMZ65 was prepared from an extract of *E. coli* transformant using Ni-NTA affinity chromatography [16,22]. SrtA enzyme activities were measured through a fluorophotometric method using synthetic peptide substrate containing the LPETG motif. The inhibitory effects, assessed using half-maximal inhibitory concentration (IC_50_) values and pIC_50_ values (negative logarithm of the IC_50_ values), of compounds **1–6** against *S. mutans* SrtA are shown in Table 2, along with that of triphasiol (IC_50_ = 25.3 μM). Triphasiol has been described as a potent inhibitor of *S. aureus* ATCC6537p-derived SrtA (IC_50_ = 34.5 μM) [21], and it exhibited strong inhibitory activity toward *S. mutans*-derived SrtA. Among the isolated compounds, 3’,3”-dihydroxy-(−)-matairesinol (**1**) exhibited the most effective inhibitory activity against *S. mutans* SrtA (IC_50_ = 16.1 μM). Analysis of SrtA inhibition by 3’,3”-dihydroxy-(−)-matairesinol (**1**) and (−)-matairesinol (**2**) indicated that the hydroxyl groups at the C-3’ and C-3” sites play important roles. Substitution of the C-3’ and C-3” hydroxyl groups of **1** through dehydroxylation (**2**) led to complete loss of activity (IC_50_ > 300 μM). 5-Hydroxyhinokitiol (**5**) and juniperone A (**6**) showed moderate inhibitory activities, with IC_50_ values of 51.7 and 62.5 μM, respectively, while quercetin (**3**) and 4,6-dihydroxy-2-methoxyacetophenone (**4**) displayed weak inhibitory activities. As SrtA inhibitors may act as anti-infective agents and disrupt bacterial infectiousness without impacting bacterial viability [4], the MICs of the isolated compounds were evaluated to identify possible effects on *S. mutans* aggregation, adhesion, and biofilm formation on solid surfaces. As shown in Table 2, no compounds evaluated herein except compound **6** (MIC = 256.9 μM) inhibited *S. mutans* OMZ65 growth (all other MICs > 300 μM).

**Table 2.** Inhibitory effects of compounds **1–6** on the activity of SrtA enzyme and bacterial growth of *S. mutans* strain OMZ65

Compound	SrtA IC_50_ (μM)	pIC_50_*	MIC (μM)**
**1**	16.1	4.79	>300
**2**	>300	-	>300
**3**	185.3	3.7	>300
**4**	151.4	3.81	>300
**5**	51.7	4.29	>300
**6**	62.5	4.20	256.9
Triphasiol	25.3	4.60	ND***
Ampicillin	ND	-	0.4

*pIC50 = −log_10_(IC_50_). **MIC means minimum inhibitory concentration. *** ND means not determined. Triphasiol and ampicillin were used as a reference inhibitor of SrtA and a standard of antibacterial drug, respectively.

### Inhibitory effects of compound 1 on saliva-induced cell aggregation

**Figure 3.**
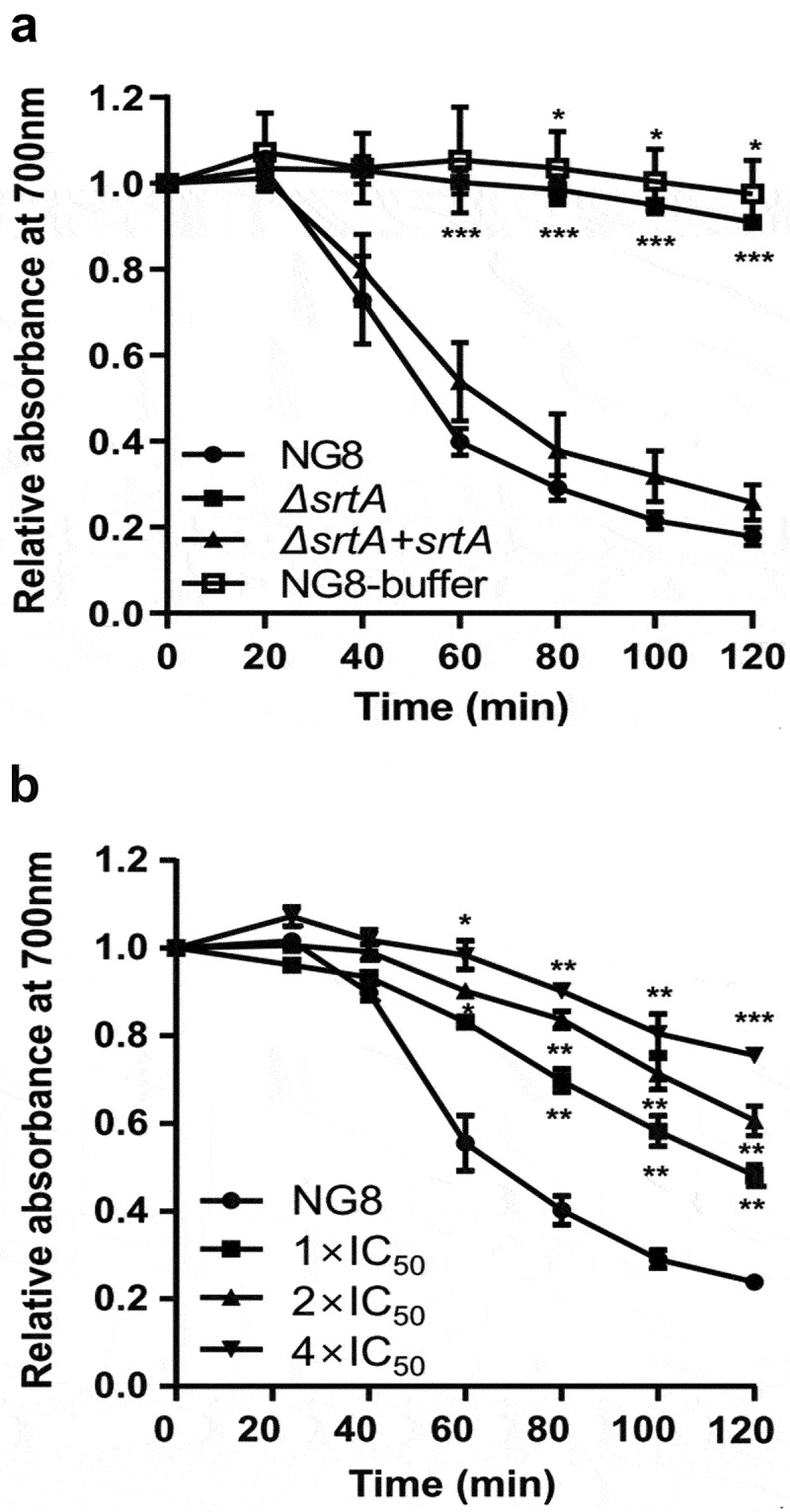
Inhibitory effects of compound **1** on saliva-induced aggregation of *Streptococcus mutans* NG8. Cells with approximate optical density of 1.0 at 700 nm were incubated aerobically at 37°C with human saliva for 2 h. **(A)** Saliva-induced aggregation of *S. mutans* NG8 (wild type), *srtA*-deletion mutant (*ΔsrtA*), and *srtA*-complemented mutant (*ΔsrtA + srtA*). NG8-buffer refers to the aggregation assay performed with *S. mutans* NG8 in the absence of saliva. **(B)** *S. mutans* NG8 treated with compound **1**. The aggregation assay was performed with *S. mutans* NG8 in the presence of 16.1 μM (1× IC_50_), 32.2 μM (2× IC_50_) and 64.4 μM (4× IC_50_) compound **1**. Each point indicates the mean ± standard deviation of three independent experiments. Results were compared using two-way analysis of variance with the post-hoc Dunnett’s test. * *p* < 0.05, ** *p* < 0.01, and *** *p* < 0.001 versus controls.

Using a combined bioactivity test, we subsequently examined the inhibitory effects of compound **1** on saliva-induced aggregation of *S. mutans* (Figure 3). The saliva-induced aggregation assay was performed using *S. mutans* strain NG8 (wild type) and isogenic *srtA*-knockout mutants. SrtA sequence alignment of NG8 (GenBank accession number: AF542085) revealed that it is identical to SrtA of *S. mutans* OMZ65 over its entire length (data not shown). Aggregation levels were measured based on the decrease of turbidity at 700 nm. As shown in Figure 3A, both the wild-type and *srtA*-complemented strains began to aggregate dramatically at 20 min, and the relative turbidity decreased by 75% at 2 h, whereas the *srtA-*deletion mutant showed no aggregation and maintained high turbidity. Interestingly, compound **1** markedly reduced the aggregation of NG8 cells in a dose-dependent manner (tested at 0×, 1×, 2×, and 4× the SrtA IC_50_) (Figure 3B).

### Inhibitory effects of compound 1 on bacterial adherence

**Figure 4.**
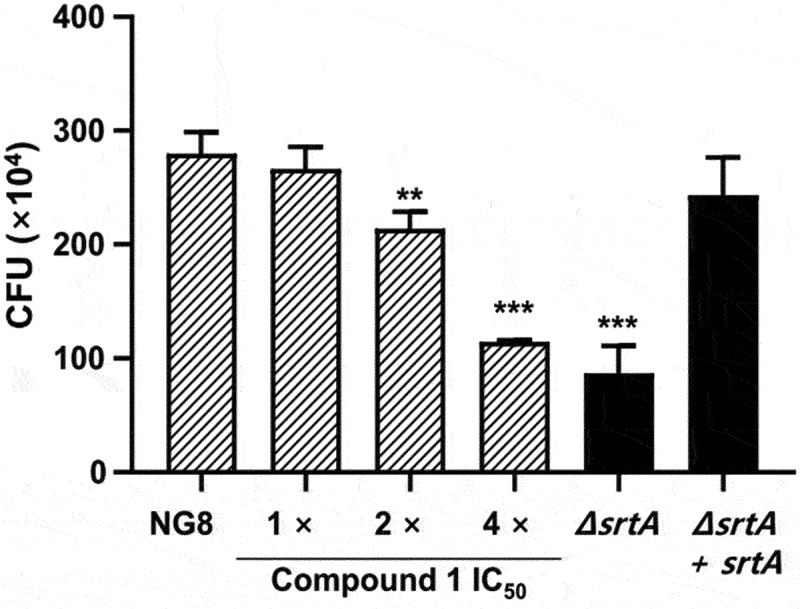
Inhibitory effects of compound **1** on *Streptococcus mutans* adherence to saliva-coated hydroxyapatite beads (S-HAs). Attachment of *S. mutans* NG8, *srtA*-deletion mutant (*ΔsrtA*), and *srtA*-complemented mutant (*ΔsrtA+srtA*) was induced for 90 min at 37°C under aerobic condition, followed by dispersal via sonication (50 W, 30s) after three washes, and then colony-forming unit counting on Mitis-Salivarius agar (3.2 mg/mL bacitracin) after incubation for 48 h at 37°C. The concentration of compound **1** ranged from 1× the IC_50_ (16.1 μM) to 4× the IC_50_ (64.4 μM). Data are presented as the mean ± standard deviation of three independent experiments (* *p* < 0.05, ** *p* < 0.01, and *** *p* < 0.001 based on Student’s *t*-test).

In the present study, the role of SrtA inhibitors in modulating cell-surface-related properties of *S. mutans* was investigated in terms of adherence. As SrtA is essential to the display of surface proteins, including adhesins in *S. mutans*, inhibition of SrtA reduces adhesion capability and aggregation of the bacteria. The adherence assay was performed on s-HAs over a concentration range of 1–4 times the IC_50_ value of compound **1**. *S. mutans* NG8 and isogenic *srtA* mutants were attached to s-HAs for 90 min, and the resulting CFUs were counted on Mitis-Salivarius agar plates. The adherence of *S. mutans* in the treatment with 1× IC_50_ (16.1 μM) of compound **1** was not significantly different from that of the control (NG8). However, in the treatment containing 4× the IC_50_ of compound **1** (64.4 μM), bacterial adhesion was markedly reduced, showing a decrease of about 60% compared to the control group (Figure 4).

### Inhibitory effects of compound 1 on biofilm formation

**Figure 5.**
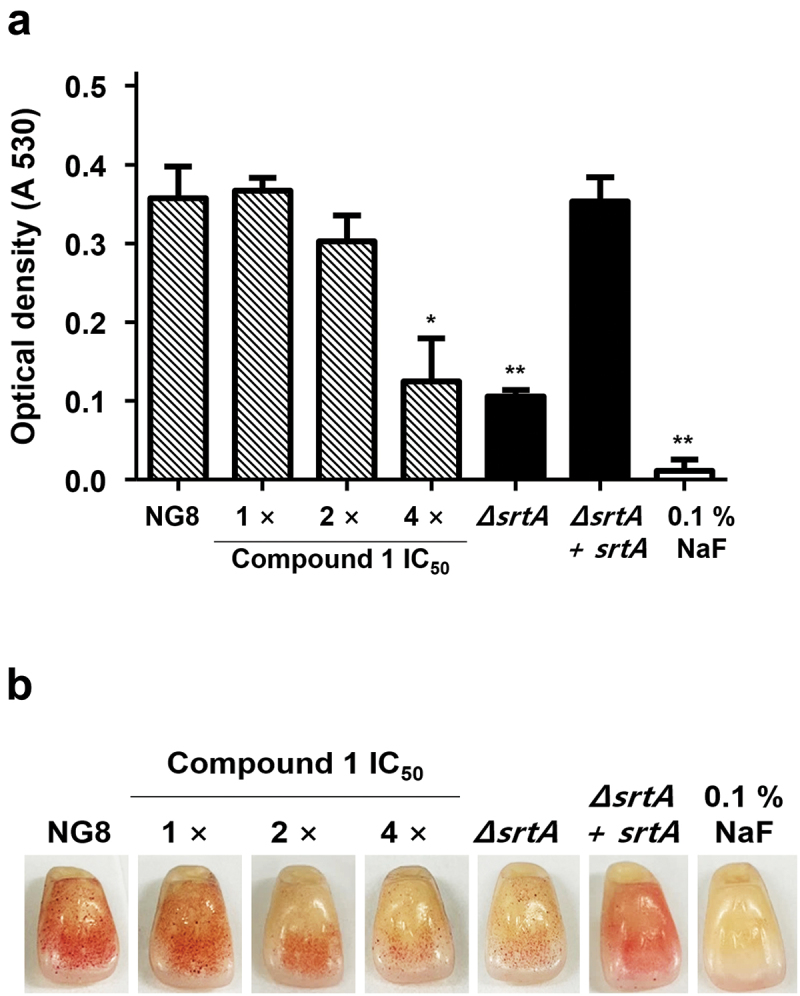
Inhibition of *Streptococcus mutans* biofilm formation by compound **1**. Biofilm formation assays were performed using a polystyrene 96-well plate **(A)** and artificial resin teeth **(B)** at 37°C for 24 h under aerobic condition. The concentration of compound **1** ranged from 1× the IC_50_ (16.1 μM) to 4× the IC_50_ (64.4 μM) and biomass of the biofilm was measured via 0.1% safranin staining. 0.1% NaF was used as a positive control. Data are presented as the mean ± SD of three independent experiments (* *p* < 0.05, ** *p* < 0.01, and *** *p* < 0.001 based on Student’s *t*-test).

Bacterial adherence and aggregation are initial steps in the biofilm formation process [10]. Through interruption of SrtA enzyme activity, biofilm formation by *S. mutans* could also be disrupted [12]. To investigate the effect of compound **1** on biofilm formation of *S. mutans*, the biofilm biomasses of NG8 and *srtA* mutants formed over 24 h on 96-well plates and resin teeth were assessed through staining with 0.1% safranin. As shown in Figure 5A, *S. mutans* attached to and aggregated on the surface of 96-well polystyrene plates, and biofilm formation was significantly reduced in the presence of compound **1** at a concentration of 4× the IC_50_ (64.4 μM), in accordance with the properties of the *srtA*-deletion mutant. We also examined biofilm formation on the surface of resin teeth, and compound **1** successfully disrupted biofilm formation at concentrations higher than 32.2 μM (Figure 5B). The positive control (0.1% NaF) also exhibited potent inhibitory effects on *S. mutans* biofilm formation. The results of these analyses suggest that compound **1**, a small-molecule inhibitor of SrtA, has potential for application to prevent dental plaque.

## Discussion

*S. mutans* uses SrtA as a tool for attaching surface proteins to the peptidoglycan cell wall, thereby supporting development of a biofilm and attachment to the tooth surface [8,10,11]. Previous studies demonstrated that *S. mutans* strains lacking SrtA exhibit a decreased ability to attach to human extracellular matrix proteins and to colonize the murine oral cavity and teeth [11,12]. Playing a key role in the attachment of surface proteins to the cell wall, SrtA became an important target in the search for anti-virulence drugs. In this study, six compounds (**1–6**) of various skeletal classes were isolated from the dried heartwood of *J. chinensis*. A novel compound designated 3’,3”-dihydroxy-(−)-matairesinol (**1**) was identified, which exhibited potent inhibitory activity toward *S. mutans* SrtA (IC_50_ = 16.1 μM) without affecting microbial viability (Table 2). The results of subsequent bioassays using compound **1** indicated that this compound inhibits *S. mutans* aggregation, adhesion, and biofilm formation on solid surfaces by inhibiting SrtA activity. The magnitudes of inhibition of adherence and biofilm formation in *S. mutans* treated with compound **1** at 4× the SrtA IC_50_ are comparable to the behaviors of the untreated *srtA*-deletion mutant (Figures 4 and 5).

Early studies demonstrated that a few compounds from Chinese traditional medicine can inhibit *S. mutans* biofilm formation by inhibiting SrtA activity [29–32]. For example, curcumin [36,37] and morin [38] show inhibitory activities against SrtA derived from *S. mutans* UA159 (IC_50_ = 10.2 μM and 27.2 μM, respectively) and interrupt biofilm formation by reducing the release of surface protein antigens I/II. A recent study using molecular docking demonstrated that myricetin is able to target the binding site of SrtA and thus inhibit SrtA activity (IC_50_ = 48.66 μM) and reduce the adhesion and biofilm formation of *S. mutans* in a sucrose-independent manner [39]. Astillbin [40], isolated from *Rhizoma Smilacis Glabrae*, inhibits SrtA (from strain ATCC25175), with an IC_50_ of 7.5 μg/mL (16.7 μM), as well as biofilm formation. Some lignans have been reported as SrtA inhibitors against *S. mutans* OMZ65, with (−)-rosmarinic acid and caffeic acid having IC_50_ values of 20.1 μM and 20.2 μM, respectively [15]. 3’,3”-Dihydroxy-(−)-matairesinol (**1**), first reported in this study, also showed inhibitory activity against *S. mutans* SrtA at a similar concentration (IC_50_ = 16.1 μM) to the substances described above, without inhibition of bacterial growth.

The active form of SrtA is required to control *S. mutans* aggregation, adherence, and biofilm formation ability, and it has been verified as a virulence factor for caries [11,12]. *S. mutans* strains lacking functional SrtA cannot adhere to solid surfaces [11]. Inhibitors of SrtA might block SrtA-mediated protein anchoring, preventing the aggregation ability of *S. mutans* cells. Based on these findings, we next conducted an assay in which saliva-induced cell aggregation was quantified through the measurement of turbidity. First, the cell aggregation capacities of *S. mutans* strain NG8 (wild type) and isogenic knockout mutants were investigated. In saliva-induced aggregation (Figure 3A), NG8 and the *srtA*-complemented mutant aggregated upon incubation with saliva, but the *srtA*-deletion mutant failed to aggregate. In addition, treatment of NG8 with 3’,3”-dihydroxy-(−)-matairesinol (**1**) markedly reduced the aggregation capacity of the bacterial cells in a dose-dependent manner (Figure 3B). These assay data suggest that the active form of SrtA is important for controlling *S. mutans* cell aggregation ability.

*S. mutans* adheres to the oral surface via two mechanisms – sucrose-independent and sucrose-dependent [41]. Sucrose-independent adhesion is mainly mediated by antigens I/II (also known as P1, Pac, and SpaP) [42–44], while sucrose-dependent adhesion is mainly mediated by glucosyltransferases (Gtfs, including GtfB, GtfC, and GtfD) [45], which also mediate interspecies coaggregation and play a critical role in the development and maturation of oral biofilms [46,47]. In addition, glucan-binding protein C (GbpC) is involved in both sucrose-dependent and sucrose-independent adherence and biofilm formation [48]. Antigens I/II and GbpC have been reported to harbor the LPXTG motif, the site of recognition and cleavage of SrtA [49]. In this study, saliva-induced cell aggregation and adherence assays on s-HAs were performed without sucrose. When *S. mutans* was incubated with 4× the IC_50_ of compound **1**, cell aggregation (Figure 3) and adherence (Figure 4) were significantly repressed. By contrast, the inhibitory effects of compound **1** on *S. mutans* biofilm formation on the surface of 96-well polystyrene plates and resin teeth were assessed under sucrose-supplemented conditions. Interestingly, *S. mutans* biofilm formation on polystyrene dishes and resin teeth was also significantly reduced following treatment with compound **1** at a concentration of 4× the IC_50_ (Figure 5). These results indicate that SrtA can bind surface proteins containing the LPXTG motif and thus initiates both sucrose-dependent and sucrose-independent adherence and biofilm formation by *S. mutans*. In addition, compound **1** blocks *S. mutans* adhesion and biofilm formation by inhibiting SrtA without being affected by sucrose.

The results from this study suggest that small-molecule inhibitors of *S. mutans* SrtA can be useful prophylactic agents for the prevention of dental plaque. However, several issues have yet to be solved. We demonstrated the ability of compound **1** to inhibit SrtA activity outside of *S. mutans* cells, but the exact effect *in vivo* remains unknown. Further study is required to clarify the relationship between the inhibition of SrtA activity and the reduction in *S. mutans* adhesion and biofilm formation and to identify the main cellular target of 3’,3”-dihydroxy-(−)-matairesinol (**1**). In addition, for SrtA inhibitors to be realized as effective drugs, direct assessment of the inactivation of SrtA and attenuation of virulence in animal models must be demonstrated. Nevertheless, small-molecule inhibitors of SrtA represent a promising approach to the effective inhibition of *S. mutans* and will benefit the management of dental caries.

## Conclusions

In the present study, chemical analysis was performed of the Korean traditional medicine *J. chinensis* to identify potential inhibitors of *S. mutans* SrtA. Bioassay-guided separation of the extract yielded six compounds (**1–6**), for which the structures were assessed through combined spectroscopic analysis. The structure of a novel compound was designated 3’,3”-dihydroxy-(−)-matairesinol (**1**). This compound displayed significant inhibitory activity against *S. mutans* SrtA (IC_50_ = 16.1 μM) without interrupting bacterial viability, whereas all other tested compounds exhibited moderate to weak inhibitory activities. The results of subsequent bioassays of compound **1** indicated that its bioactivity is associated with the inhibition of SrtA-mediated *S. mutans* aggregation, adhesion, and biofilm formation on the surface of resin teeth. Our findings suggest that small-molecule inhibitors of *S. mutans* SrtA may be useful for the prevention of dental caries and treatment of dental microbial diseases.

## Supplementary Material

Supplemental Material
