# Supplementary material for: Inhibition of Streptococcus mutans adhesion and biofilm formation with small-molecule inhibitors of sortase A from Juniperus chinensis
Source: J Oral Microbiol. 2022 Jun 14;14(1):2088937. doi: 10.1080/20002297.2022.2088937 (PMC9225741; doi:10.1080/20002297.2022.2088937)
Supplement: Supplemental Material [file ZJOM_A_2088937_SM2754.docx]

***Supporting Information***

**Inhibition of *Streptococcus mutans* adhesion and biofilm formation with small-molecule inhibitors of sortase A from *Juniperus chinensis***

Anonymous

**Contents**

**Figure S1.** The ^1^H NMR spectrum of 3',3''-dihydroxy-(-)-matairesinol (**1**) (600 MHz, DMSO-*d*_6_) S3

**Figure S2.** The ^13^C NMR spectrum of 3',3''-dihydroxy-(-)-matairesinol (**1**) (150 MHz, DMSO-*d*_6_) S4

**Figure S3.** The HSQC spectrum of 3',3''-dihydroxy-(-)-matairesinol (**1**) (600 MHz, DMSO-*d*_6_) S5

**Figure S4.** The COSY spectrum of 3',3''-dihydroxy-(-)-matairesinol (**1**) (600 MHz, DMSO-*d*_6_) S6

**Figure S5.** The HMBC spectrum of 3',3''-dihydroxy-(-)-matairesinol (**1**) (600 MHz, DMSO-*d*_6_) S7

**Figure S6.** The NOESY spectrum of 3',3'-dihydroxy-(-)-matairesinol (**1**) (500 MHz, DMSO-*d*_6_) S8

**Figure S7.** The HR-ESI-MS data of 3',3''-dihydroxy-(-)-matairesinol (**1**) S9

**Table S1.** The deviations with literature of ^13^C NMR chemical shifts of **3** S10

**Table S2.** The deviations with literature of ^13^C NMR chemical shifts of **2,4-5** S11


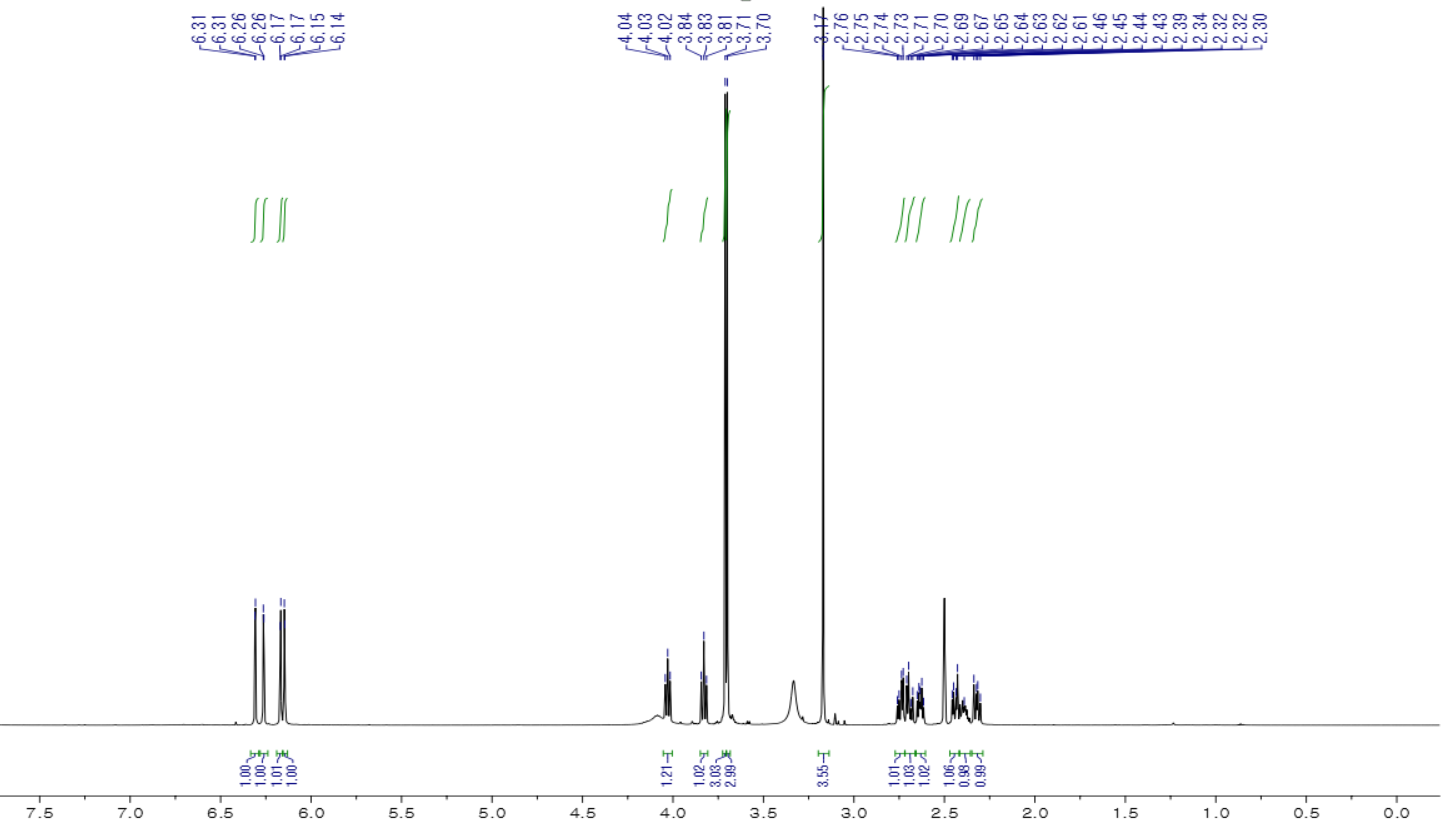


**Figure S1**. The ^1^H NMR spectrum of 3',3''-dihydroxy-(-)-matairesinol (**1**) (600 MHz, DMSO-*d*_6_)


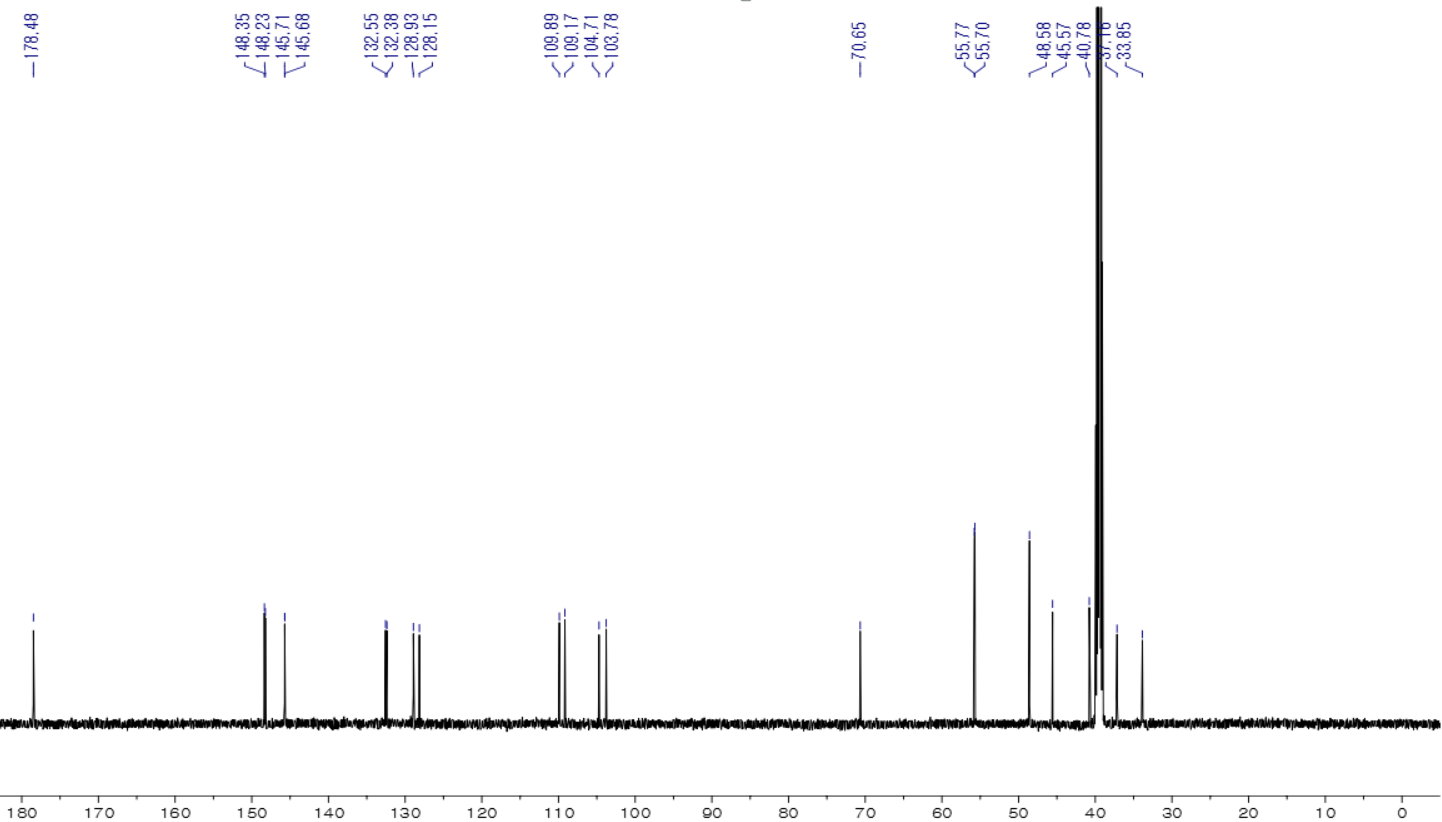


**Figure S2**. The ^13^C NMR spectrum of 3',3''-dihydroxy-(-)-matairesinol (**1**) (150 MHz, DMSO-*d*_6_)


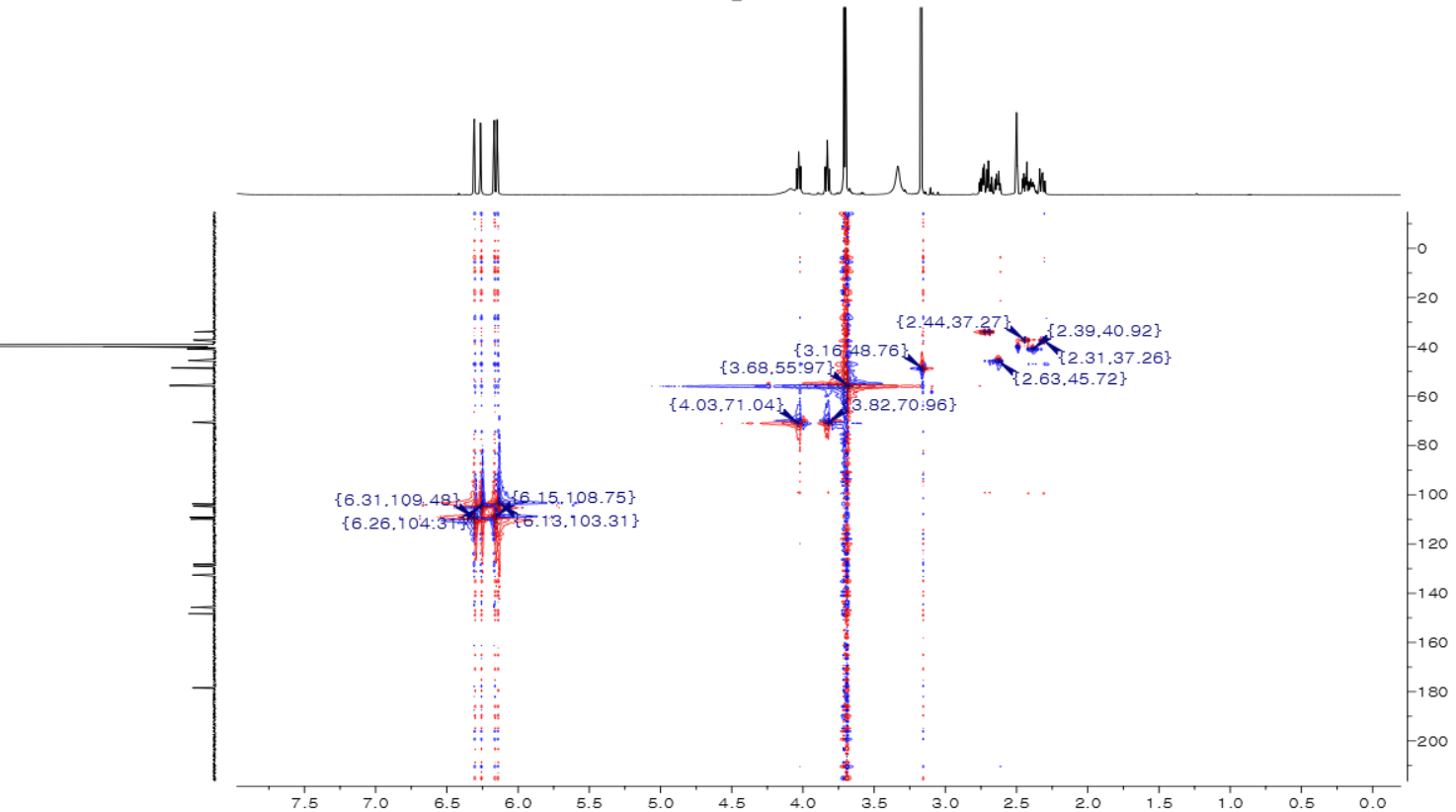


**Figure S3**. The HSQC spectrum of 3',3''-dihydroxy-(-)-matairesinol (**1**) (600 MHz, DMSO-*d*_6_)


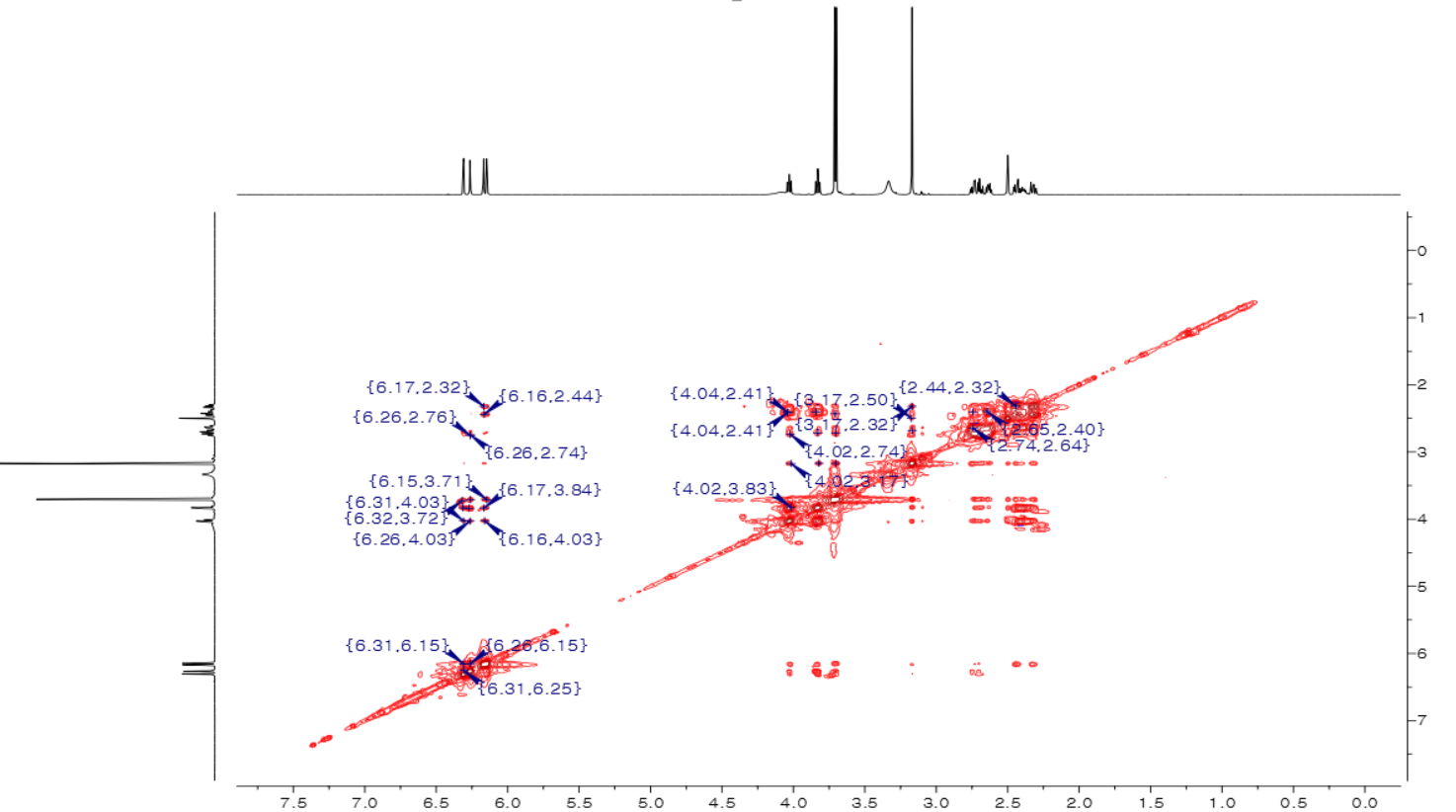


**Figure S4**. The COSY spectrum of 3',3''-dihydroxy-(-)-matairesinol (**1**) (600 MHz, DMSO-*d*_6_)


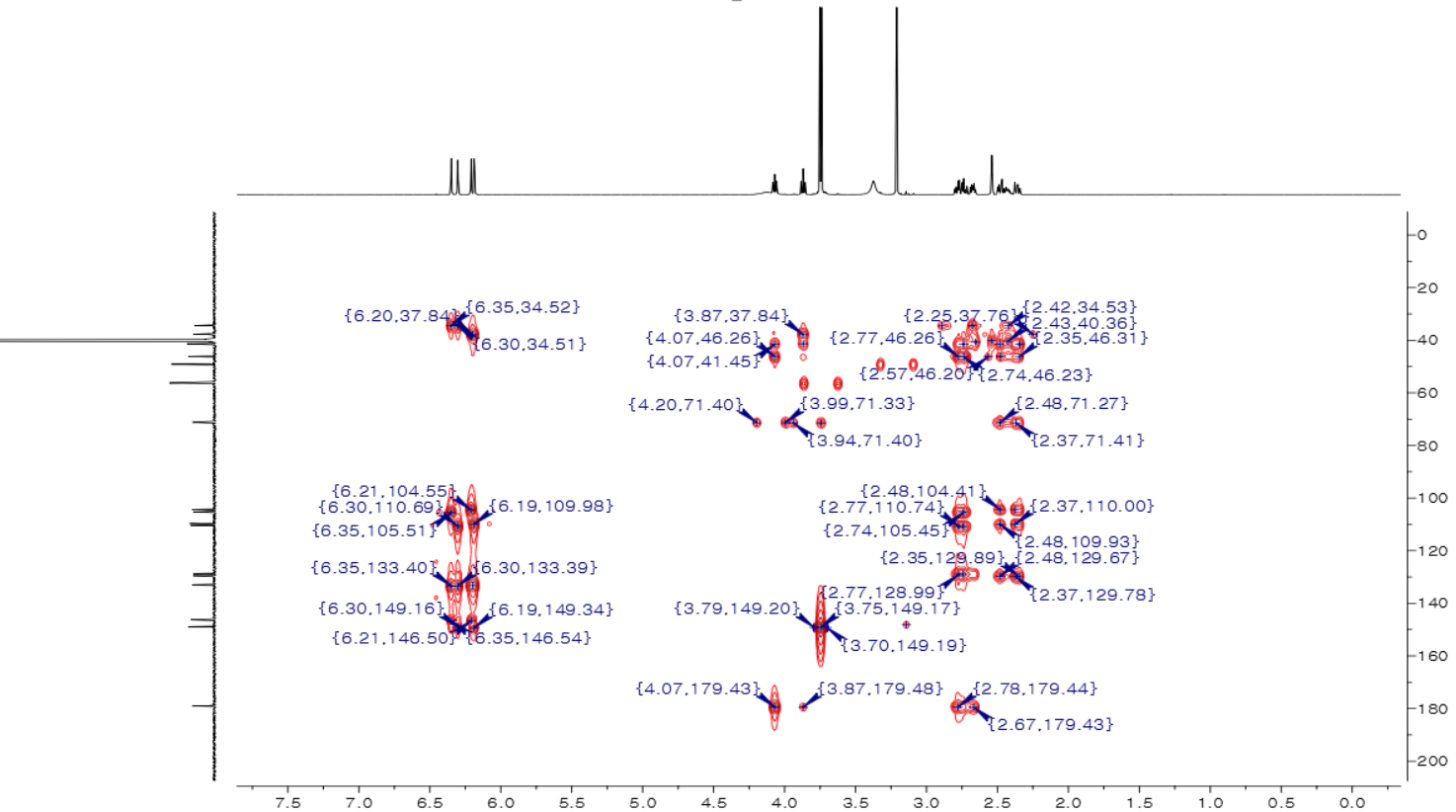


**Figure S5**. The HMBC spectrum of 3',3''-dihydroxy-(-)-matairesinol (**1**) (600 MHz, DMSO-*d*_6_)


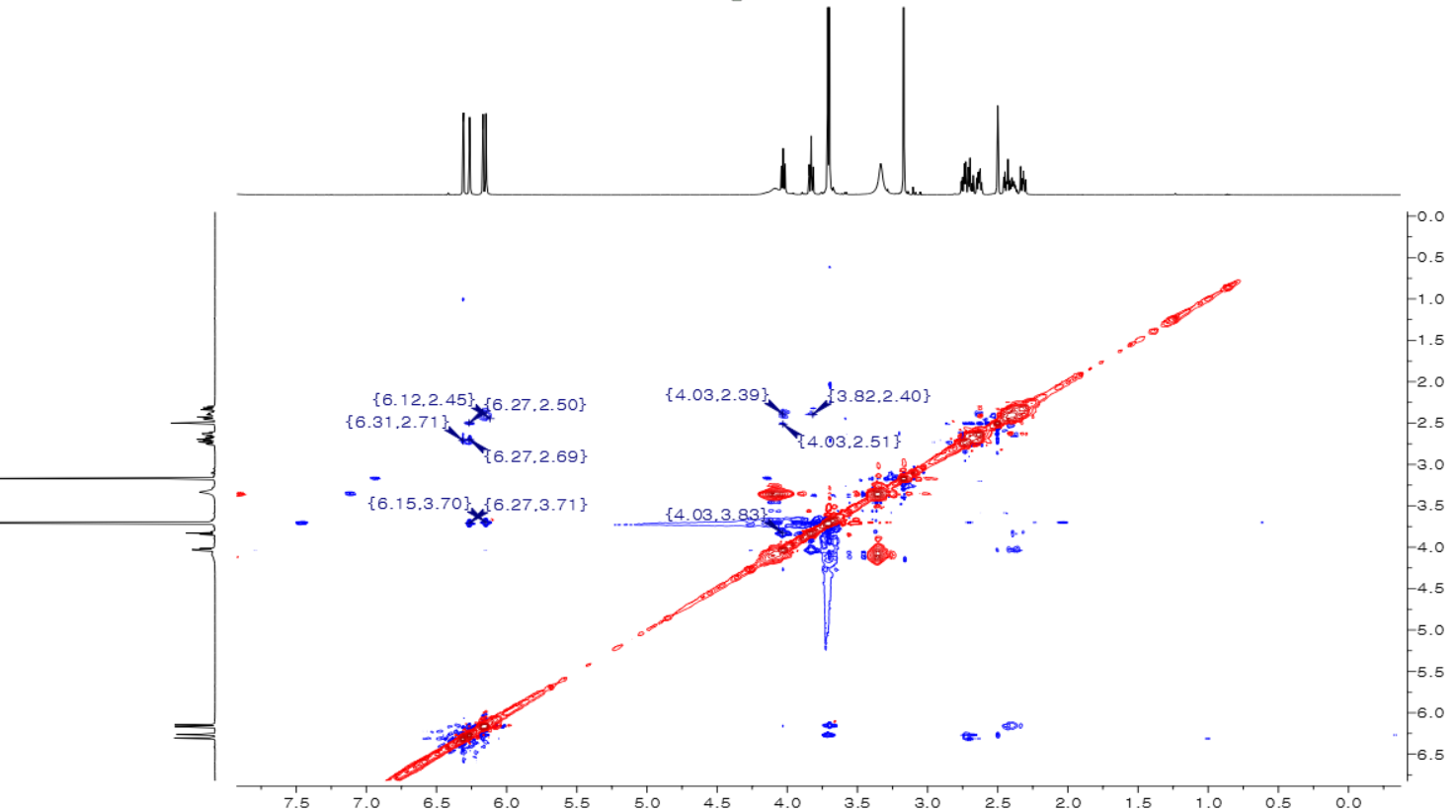


**Figure S6**. The NOESY spectrum of 3',3''-dihydroxy-(-)-matairesinol (**1**) (500 MHz, DMSO-*d*_6_)


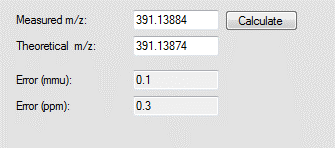


**Figure S7**. The HR-ESI-MS data of 3',3''-dihydroxy-(-)-matairesinol (**1**)

**Table S1.** The deviations with literature of ^13^C NMR chemical shifts of **3**

| **Quercetin** | | |
| --- | --- | --- |
| **Ref. ^1^** | **3** | **Dev.** |
| 177.4 | 177.9 | -0.5 |
| 165.8 | 167.1 | -1.3 |
| 162.5 | 163.1 | -0.6 |
| 158.4 | 159.0 | -0.6 |
| 148.7 | 149.5 | -0.8 |
| 148.2 | 148.6 | -0.4 |
| 146.4 | 146.5 | -0.1 |
| 137.5 | 137.9 | -0.4 |
| 124.2 | 124.9 | -0.7 |
| 121.8 | 122.4 | -0.6 |
| 116.4 | 116.9 | -0.5 |
| 116.3 | 116.7 | -0.4 |
| 104.8 | 105.0 | -0.2 |
| 99.5 | 100.3 | -0.8 |
| 94.6 | 95.3 | -0.7 |

^1^ Jung K, Jeon JS, Ahn MJ, Kim CY, Kim J. Preparative isolation and purification of flavonoids from *Pterocarpus saltalinus* using centrifugal partition chromatography. J Liq Chromatogr Relat Technol. 2012;35:2462-2470. doi: 10.1080/10826076.2011.633680.

**Table S2.** The deviations with literature of ^1^H NMR and ^13^C NMR chemical shifts **2, 4-5**

| **Matairesinol** | | | **4,6-dihydroxy-2-methoxyacetophenone** | | | **5-hydroxyhinokitiol** | | |
| --- | --- | --- | --- | --- | --- | --- | --- | --- |
| **Ref.^1^** | **2** | **Dev.** | **Ref.^2^** | **4** | **Dev.** | **Ref.^3^** | **5** | **Dev.** |
| 178.7 | 178.4 | 0.3 | 202.2 | 202.0 | 0.2 | 7.44 | 7.46 | -0.02 |
| 147.6 | 147.4 | 0.2 | 166.3 | 166.1 | 0.2 | 7.17 | 7.19 | -0.02 |
| 147.5 | 147.3 | 0.2 | 165.2 | 164.9 | 0.3 | 7.06 | 7.08 | -0.02 |
| 145.2 | 145.0 | 0.2 | 163.4 | 163.1 | 0.3 | 3.56 | 3.56 | 0.00 |
| 145.0 | 144.9 | 0.1 | 104.6 | 104.3 | 0.3 | 1.22 | 1.23 | -0.01 |
| 129.2 | 129.5 | -0.3 | 95.6 | 95.3 | 0.3 | 1.22 | 1.22 | 0.00 |
| 129.0 | 128.8 | 0.2 | 91.3 | 91.1 | 0.2 |  |  |  |
| 121.7 | 121.5 | 0.2 | 55.8 | 55.6 | 0.2 |  |  |  |
| 120.8 | 120.6 | 0.2 | 32.6 | 32.4 | 0.2 |  |  |  |
| 115.5 | 115.3 | 0.2 |  |  |  |  |  |  |
| 115.4 | 115.2 | 0.2 |  |  |  |  |  |  |
| 113.5 | 113.4 | 0.1 |  |  |  |  |  |  |
| 112.7 | 112.6 | 0.1 |  |  |  |  |  |  |
| 70.8 | 70.7 | 0.1 |  |  |  |  |  |  |
| 55.6 | 55.5 | 0.1 |  |  |  |  |  |  |
| 55.6 | 55.4 | 0.2 |  |  |  |  |  |  |
| 45.8 | 45.6 | 0.2 |  |  |  |  |  |  |
| 41.0 | 40.9 | 0.1 |  |  |  |  |  |  |
| 37.0 | 36.9 | 0.1 |  |  |  |  |  |  |
| 33.8 | 33.7 | 0.1 |  |  |  |  |  |  |

^1^ Li Z, Sha Y, Chen L, Hu J, Wang S, Meng D, Wang N. Two lignans from the fine roots of *Cunninghamia lanceolate*. Chem Nat Compd. 2013;49(2):229–231. doi: 10.1007/s10600-013-0568-2

^2^ Wei Y, Tang J, Conga X, Zeng X. Practical metal-free synthesis of chalcone derivatives via a tandem cross-dehydrogenative-coupling/elimination reaction. Green Chem. 2013;15:3165–3169. doi: 10.1039/C3GC41403E

^3^ Takeshita H, Kusaba T, Mori A. Intentional syntheses of utahin, a conifer constituent of A C_20_-ditroponofuran, via the oxidative condensation. Chern Lett. 1983;12(9):1371-1372. doi: 10.1246/cl.1983.1371
